# Supplementary material for: Perilipin 5 and Lipocalin 2 Expression in Hepatocellular Carcinoma
Source: Cancers (Basel). 2019 Mar 19;11(3):385. doi: 10.3390/cancers11030385 (PMC6468921; doi:10.3390/cancers11030385)
Supplement: Supplementary file 1 [file cancers-11-00385-s001.pdf]

cancers

MDPI

Supplementary Materials: Perilipin 5 and Lipocalin 2 Expression in Hepatocellular Carcinoma

Anastasia Asimakopoulou, Mihael Vucur, Tom Luedde, Silvia Schneiders, Stavroula Kalampoka, Thomas S. Weiss and Ralf Weiskirchen

Table S1. Characteristics of HCC patients (*n* = 17).

HCC-Patient #	1	2	3	4	5	6	7	8	9	10	11	12	13	14	15	16	17
**Age**	85	51	78	76	33	68	64	72	63	46	69	65	49	82	59	79	64
**Sex**	f	f	f	f	f	m	m	m	m	m	m	f	m	m	m	m	m
**Height (cm)**	146	160	160	175	155	94	170	177	174	180	181	155	169	171	187	168	172
**Weight (kg)**	56	58	71	73	59	168	57	84	89	106	93	89	76	94	106	110	68
**AFP (μg/L)**	11.2	3.4	11.1	11.4	10.6	4.5	246	ND	2.9	11.7	5.1	379.2	141	5.8	2.3	1.5	7.2
**AP (U/L)**	63	90	357	237	69	72	ND	243	103	124	57	ND	97	47	69	105	102
**Albumin (g/L)**	35.4	42.1	19	26.5	ND	ND	10	12.2	31.5	25.9	ND	ND	ND	ND	ND	ND	ND
**Bilirubin (mg/dL)**	0.3	0.4	1.1	0.5	0.4	0.6	0.6	0.4	0.15	0.9	1.13	0.68	0.73	0.8	0.9	0.4	0.5
**AST (U/L)**	29	39	82	19	14	38	45	145	22	57	23	42	75	16	55	17	50
**ALT (U/L)**	17	27	54	51	22	62	67	56	34	47	31	29	145	18	64	21	45
**GGT (U/L)**	89	167	124	188	64	108	287	201	55	135	16	146	346	62	203	69	188
**PTT (s)**	29.2	27.5	28.4	27.3	27.8	33.9	30.2	32.7	27.5	38.8	40.4	30.2	34	29.9	25.7	29.2	32.8
**Quick (%)**	89	92	95	>100	98	97	107	>100	>100	78	92	87	97	>100	92	99	>100
**ChE (U/L)**	8529	8523	6165	12,542	9085	10,022	4345	8818	15,861	7848	4738	5262	7498	10,008	14,331	9811	10,860
**Tumour Tissue**	HCC	HCC	HCC	HCC	HCC	HCC	HCC	HCC	HCC	HCC	HCC	HCC	HCC	HCC	HCC	HCC	HCC
**Tumour Size (cm)**	11.6	2.6	18.9	8.8	5.5	5.1	25	18.3	2.5	4	0.9	3.5	9.5-10.5	4.1	9.8	8.4	4
**Grading ***	2	2	3	2	1	2	2	2	2	2	3	2	2	2	2	1	2
**Tumour Size**	2	1	4	3a	1	1	3a	2	2	1	1	2	3a	1	1	1	2
**Regional Lymph Node Infestation**	0	0	1	0	0	0	1	0	0	0	0	0	0	0	0	0	0
**Venous invasion**	1	0	2	0	0	0	1	1	1	0	0	0	0	0	0	0	1
**Distance to Resection Edge**	0	0	0	1	0	0	0	0	2	0	0	0	0	0	0	0	0
**Fibrosis ****	2	4	0	0	0	2	0	2	0	4	0	4	2	3 & 4	2	4	3
**Inflammation *****	1	4	4	4	4	0	4	1	1	4	4	1	4	1	1	1	1
**Diabetes Mellitus**	yes	no	yes	no	no	no	no	yes	yes	no	yes	yes	no	yes	yes	no	no
**HBV**	no	no	no	no	no	no	no	no	no	no	no	no	no	no	no	no	no
**HCV**	no	yes	no	no	no	no	no	no	no	no	no	no	yes	no	no	no	no
**HIV**	no	no	no	no	no	no	no	no	no	no	no	no	no	no	no	no	no

consecutive patient number, * Grading was done for tumor size, regional lymph node infestation, venous invasion, and distance to resection edge. ** Fibrosis scoring: 0, no fibrosis; 1, zone 3 perisinusoidal/pericellular fibrosis; focally or extensively present; 2, zone 3 perisinusoidal/pericellular; fibrosis with focal or extensive periportal fibrosis; 3, zone 3 perisinusoidal/pericellular; fibrosis and portal fibrosis with focal or extensive bridging fibrosis; 4, cirrhosis. *** Inflammation scoring: 0 = no foci; 1 = <2 foci; 2 = 2–4 foci; 3 = >4 foci; 4 = yes but not further specified. AP, alkaline phosphatase; AST, aspartate transaminase; ALT, alanine aminotransferase; GGT, γ-glutamyltransferase, PTT, partial thromboplastin time; ChE, cholinesterase, HBV, hepatitis B virus; HCV, hepatitis C virus, HIV, human immunodeficiency virus; f, female; m, male; ND, not determined.

Table S2. Characteristics of Non-HCC patients (*n* = 10).

Non-HCC-Patient #	1	2	3	4	5	6	7	8	9	10
Age	62	72	54	51	77	72	57	74	57	67
Sex	m	m	f	f	m	f	m	f	m	f
Height (cm)	186	175	163	159	171	170	167	170	171	164
Weight (kg)	157	82	70	79	64	80	84	83	78	55
AFP (µg/L)	5.5	4.6	0.9	3.3	ND	5.6	ND	7.8	4.1	ND
AP (U/L)	91	84	122	93	233	107	120	98	81	148
Albumin (g/L)	38.2	23.6	19.7	18.8	19.7	27	ND	28.8	20.6	16.3
Bilirubin (mg/dL)	0.3	0.4	0.3	0.2	0.3	0.3	0.4	0.6	0.7	0.43
AST (U/L)	19	20	29	18	23	14	20	16	24	22
ALT (U/L)	24	29	22	18	15	16	18	22	36	21
GGT (U/L)	56	31	151	43	495	46	65	76	50	149
PTT (s)	28.7	35.4	27.9	27	37.7	28.1	23.7	26.3	27.3	31.9
Quick (%)	>100	72	>100	>100	96	>100	>100	94	99	97
ChE (U/L)	10,971	11,617	12,377	12,419	7444	15,331	6383	11,601	12,476	10,403
Resection Tumour Tissue	Metastasis from colon carcinoma	Metastasis from colon carcinoma	Metastasis from rectal carcinoma	Metastasis from colon carcinoma	Metastasis from rectal carcinoma	Metastasis from colon carcinoma	Metastasis from rectal carcinoma	Metastasis from rectal carcinoma	Metastasis from rectal carcinoma	Metastasis from rectal carcinoma
Fibrosis *	0	0	ND	0	ND	1	1	0	0	ND
Inflammation **	ND	0	ND	0	1	ND	1	ND	0	ND
Diabetes Mellitus	no	no	no	no	no	no	no	no	no	no
HBV	no	no	no	no	no	no	no	no	no	no
HCV	no	no	no	no	no	no	no	no	no	no
HIV	no	no	no	no	no	no	no	no	no	no

consecutive patient number, * Fibrosis scoring: 0, no fibrosis; 1, zone 3 perisinusoidal/pericellular fibrosis; focally or extensively present; 2, zone 3 perisinusoidal/pericellular; fibrosis with focal or extensive periportal fibrosis; 3, zone 3 perisinusoidal/pericellular; fibrosis and portal fibrosis with focal or extensive bridging fibrosis; 4, cirrhosis. ** Inflammation scoring: 0 = no foci; 1 = <2 foci; 2 = 2–4 foci; 3 = >4 foci; 4 = yes but not further specified. AP, alkaline phosphatase; AST, Aspartate transaminase; ALT, alanine aminotransferase; GGT, γ-glutamyltransferase, PTT, partial thromboplastin time; ChE, cholinesterase, HBV, hepatitis B virus; HCV, hepatitis C virus, HIV, human immunodeficiency virus; f, female; m, male; ND, not determined.

© 2019 by the authors. Licensee MDPI, Basel, Switzerland. This article is an open access article distributed under the terms and conditions of the Creative Commons Attribution (CC BY) license (http://creativecommons.org/licenses/by/4.0/)
